# Interesting to Physicians

**Published:** 1857-09

**Authors:** 


*Interesting to Physicians.*—The Supreme Court of the Eighth Judicial District of New York, has decided that the action of the Erie County Medical Society, in expelling Dr. John D. Hill, for taking the office of physician to the county almshouse, at a rate less than the price fixed by their tariff, was illegal, and Dr. Hill must be restored to the privileges of Membership. The Court decided that the tariff of prices was unauthorized, and contrary to public policy and law.—*Western Lancet.*

